# Exploring the Positive User Experience Possibilities Based on Product Emotion Theory: A Beverage Unmanned Retail Terminal Case

**DOI:** 10.3389/fpsyg.2022.889664

**Published:** 2022-06-16

**Authors:** Enguo Cao, Yanjun Duan, Jinzhi Jiang, Hui Peng, Weifeng Hu

**Affiliations:** ^1^Intelligent Interaction Design Laboratory, School of Design, Jiangnan University, Wuxi, China; ^2^School of Art and Design, Zhengzhou University of Light Industry, Zhengzhou, China

**Keywords:** user experience design, unmanned retail terminal, product emotion, positive user experiences, future possibility

## Abstract

Since the last century, user experience has been regarded as a key concept in the process of product and service design. With the development of positive psychology, the transformation from negative to positive user experience has also taken place in the field of user experience; it emphasizes exploring the future possibility of positive user experience rather than just solving existing problems. Based on the research and analysis of existing literature, this study makes it clear that positive user experience research should be based on the “positive experience,” and arousing a positive emotion is conducive to improving positive user experience. On this basis, the product emotion theory is applied to the analysis process of “positive experience.” Through word frequency screening, thematic analysis, and correlation calculation, the relationship between product stimulus (object, activity, and identity) and user concern (goal, attitude, and standard) based on positive “user comments” is constructed, and positive user experience is understood from multiple levels. Based on the comment score, the positive user experience interval is divided in order to clarify the improvement direction. Finally, taking the “Angel Orange” unmanned retail terminal as an example, this study carried out an empirical analysis. As an exploratory study, this study can provide some insights into the quantitative research process of positive user experience design that evokes positive emotions from a user’s “positive experience” story.

## Introduction

With the development of user experience in various disciplines (especially design) and the upgrading and transformation of user needs and expectations, users’ attention to the experience process is reflected not only in practical (availability and function) but also in hedonic (emotion and demand satisfaction) (Kaasinen et al., 2015; Müller et al., 2016; Bettiga et al., 2020) aspects. Moreover, hedonic aspects and emotions are often mentioned at the same time (Mekler and Hornbæk, 2016; Wang, 2017). The traditional research experience of human–computer interaction (HCI), interaction design, and industrial design tends to adopt a problem-driven design in most cases. It emphasizes that design is an activity to eliminate problems (Hassenzahl, 2010; Li and Wu, 2020). However, avoiding negative effects is not equal to a positive user experience (Desmet and Hassenzahl, 2012). A positive user experience design is rooted in human practice and needs, and it emphasizes a possibility-driven design (Jiminez et al., 2014; Tamminen and Moilanen, 2016). The purpose of a possibility-driven design is to change from a neutral to a positive state and explore future development potential and prospects. The latter has more direct potential to produce a valuable design, which makes the user happy (Desmet and Hassenzahl, 2012; Alenljung et al., 2019).

After clarifying that arousing a positive emotion is conducive to improving positive user experience; this study explores positive user experience through positive “user experience” in specific situations based on the product emotion theory and points out the promotion direction of a positive user experience design. Specifically, nouns in positive user comments are mapped to product stimuli (object, activity, and identity) in the product emotion theory, adjectives are mapped to user concerns (goal, attitude, and standard), and a multi-level correlation between them is constructed. Then, the user score is introduced to explore the positive user experience from the possibility and feasibility and used to guide the design practice.

The rest of this article is organized as follows. The “Literature Review” section gives a comprehensive review of relevant studies. The specific design process and method are described in the section “Exploring the Positive User Experience.” In the “Empirical Research” section, “Angel Orange”—beverage unmanned retail terminal—is discussed, and the positive user experience promotion direction is pointed out to verify the feasibility of this design process. The “Conclusion” section points out the relevant research results. In the “”Discussion” section, the contribution and future work of this study are summarized.

## Literature Review

This section summarizes the relevant literature on positive user experience, provides a comprehensive review of the relevant research, and finally defines the models/methods of a positive user experience design.

### Positive User Experience Literature Research

A positive design is adopted from positive psychology (Günay and Erbuğ, 2015; Diefenbach, 2018a). Desmet and Pohlmeyer first proposed a “positive design,” which emphasizes improving individual subjective wellbeing and bringing people a happy and meaningful life by creating and improving products or services (Desmet and Pohlmeyer, 2013). Compared with the general experience design, the “positive” of the positive user experience design has three meanings.

The first meaning refers to positive psychology, which is different from traditional psychology, that focuses on the negative aspects of problems (Seligman and Csikszentmihalyi, 2014). Positive psychology emphasizes positive factors, such as happiness, and positive qualities, such as virtue and potential (Pawelski, 2016; Ryff, 2022). Among them, positive emotions and user experiences are often discussed and studied in the field of design, including happiness, subjective wellbeing, and love (Dodge et al., 2012; Baños et al., 2017; Diefenbach, 2018b).

The second meaning refers to positive emotions. A positive user experience design emphasizes on improving individual subjective wellbeing (Desmet et al., 2013; Wu et al., 2021). A positive emotion is an important element of subjective wellbeing (Krueger and Stone, 2014; Diener et al., 2018), a direct source designed for pleasure (Lyubomirsky and Layous, 2013; Wu et al., 2019), and is closely related to the realization of personal wellbeing (Yoon et al., 2020). In other words, a person who often experiences positive emotions is more likely to have a positive evaluation or satisfaction with life (Mens et al., 2021). Therefore, the “positive” of a positive user experience design represents a positive emotion triggered in the experience process to a certain extent.

The last meaning refers to the positive transformation of “from negative to positive” user experience, which is embodied in the transformation from a problem-driven to a possibility-driven design. A problem-driven design aims to reduce or solve negative factors that may lead to a negative experience. Then, the transition from a negative state to a neutral state is realized. A possibility-driven design method focuses on supporting existing possibilities and creating new possibilities to realize the transformation from a neutral state to a positive state. This transformation is of great significance in the user experience research field, emphasizing that a positive and possibility-oriented approach must be taken in the design (Desmet and Hassenzahl, 2012; Desmet and Pohlmeyer, 2013).

### Positive User Experience Design Method/Model

The positive user experience design research started late and is still in the stage of exploration and development. Jaramillo, Pohlmeyer, and others jointly wrote the “Positive Design Reference Guide.” The guide is divided into two chapters: psychology and design (Jaramillo et al., 2015). The models and frameworks related to a positive user experience design are listed in this guide. Generally speaking, the specific method/model still revolves around the three meanings of “positive.”

For example, starting from positive psychology, the different functions of a positive design, to the five elements of the PERMA model (i.e., positive emotion, engagement, relationships, meaning, and accomplishment), a design wellbeing matrix is proposed. Moreover, the PERMA model has been used in many academic studies (Seligman, 2018; Donaldson et al., 2021; Farmer and Cotter, 2021).

**FIGURE 1:**
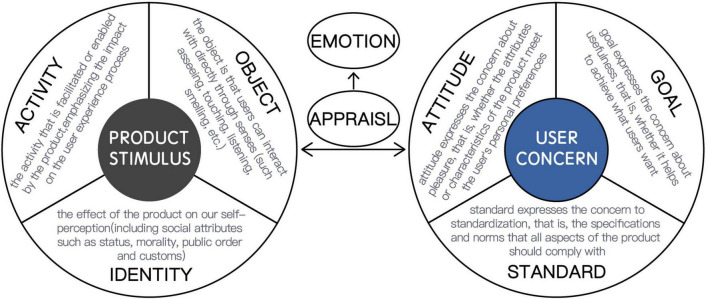
Product emotion theory parameter interpretation.

Using positive emotions, existing studies tend to appraise or distinguish different positive emotions that may be involved in the experience process. For example, the need of designers to assess user emotions about products led to the development of refined assessment tools, such as PrEmo, that contain seven animated characters, each portraying a distinct positive emotion (Laurans and Desmet, 2017). The positive emotional granularity card helps designers communicate differences between positive emotions by depicting definitions of emotions, underlying causes, and visuals of expressive manifestations (Yoon et al., 2017). Overall, 17 positive experience categories are introduced, and their patterns of eliciting conditions are described (Zeiner et al., 2018). It can be seen that these existing related studies are devoted to how users express their positive emotions to designers, with the research results depending on the user’s own emotional expression ability to a great extent. With the intention to provide users with pleasurable experiences, both design researchers and practitioners have attempted to make the process of designing for positive emotions actionable and systematic. Designers can benefit from knowing under what conditions different positive emotions arise to evoke certain positive emotions. The general view is that the emotion generated in the interaction process is not only determined by the product itself but also related to personal concerns and specific situations. Based on this, the generation process of product emotions from products and users is according to the appraisal theory (Desmet, 2003). It emphasizes that the emotion generation process is universal. Once this potential process is identified, the emotional results can be understood and even predicted. The model defines four parameters related to the emotion process: user concern, product stimulus, appraisal, and emotion (divided into negative and positive). The first three parameters determine whether to cause a certain emotion. Furthermore, the user concern is divided into goals, attitudes, and standards, and the product stimulus is divided into object, activity, and identity (Desmet, 2010; Ozkaramanli and Desmet, 2012), as shown in Figure 1.

From the perspective of transformation of “from negative to positive” user experience, it is emphasized that a positive experience design should start from the user’s “positive experience” story because the story can provide not only design problems but also valuable information about design possibilities; for example, Positive Practice Canvas—a design tool that helps designers systematically gather nuanced insights into everyday “positive experience” stories and design for them (Klapperich et al., 2018).

In general, a positive experience design emphasizes taking “positive experience” as the starting point, paying attention to the unique impact of positive emotions on positive experience and finally improving subjective wellbeing. Human emotions are a holistic experience that involves a wide range of behavioral, psychological, and physiological aspects (Yoon et al., 2020). In particular, there is now an increasing emphasis on the unique functions of positive emotions in design research and human–computer interaction (Calvo and Peters, 2014). There are considerable pieces of evidence showcasing that positive emotions play critical roles in perception, thoughts, behavior, and, by extension, improved wellbeing (Tugade et al., 2014). Products that can provide positive emotional experiences in the entire cycle of the user, i.e., product relationship can have a greater and long-lasting economic impact. In brief, designing a product or service that attracts or stimulates the positive emotions is beneficial to eliciting a positive user experience (Kim et al., 2018).

## Positive User Experience Design Process Based on the Product Emotion Theory

**FIGURE 2:**
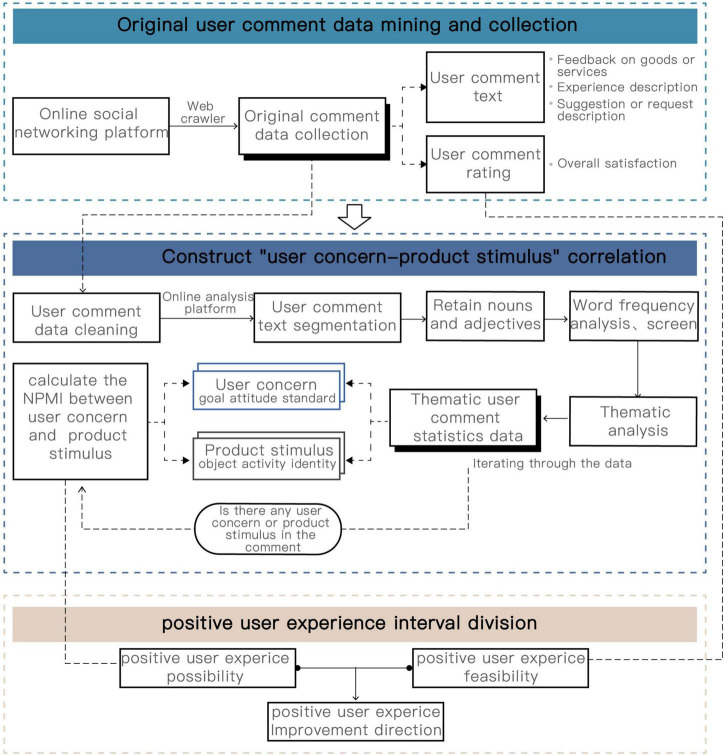
Positive user experience design exploration process.

This section proposes a positive user experience design exploration process based on the product emotion theory, as shown in Figure 2. It mainly includes obtaining the user’s “positive experience” story, constructing the correlation between user concerns and product stimuli based on the product emotion appraisal theory and importing the user evaluation results to divide the positive user experience interval.

### Acquiring User “Positive Experience” Story Based on Text Mining

According to previous research, a positive user experience design emphasizes taking positive and meaningful stories as the starting point (Korhonen et al., 2010). Conventional methods for measuring user experience, such as user interviews, focus groups, field surveys, user logs, and scale calculations, are time-consuming, laborious, and limited to collecting specific information (Pimenta et al., 2020), and the cost of obtaining a large amount of data is high. Therefore, this study uses the online user comments from social network platforms as the original data. These data are derived from active sharing based on users’ own experience, which is subjective and timely and has a large amount of data (Yang et al., 2019). Online user comments, one of the ways for users to freely express their experiences, could provide valuable information about future possibilities and user emotions (Bacha, 2018). Moreover, online user comment data contain comment text, scores, pictures, videos, and other information (Qian et al., 2019), which can buffer the false comment data problem to a certain extent. The target web page data can be easily obtained through the web crawler technology to form an original user comment database (Khan et al., 2020).

### Constructing the “User Concern–Product Stimulus” Correlation Through Data Analysis

The original user comment data are cleaned and filtered from the two aspects of comment scoring and text content identification. The filtered data analysis process is as follows.

The first step is word segmentation. The filtered data is imported into the online word segmentation platform; pronouns, adverbs, prepositions, and other words with no practical meaning are removed, and nouns and adjectives are kept because nouns represent the unified names of people, things, things, places, or abstract concepts, which are reflected in user comments as various product elements involved in the user experience process, and are consistent with the content of the product stimulus. Adjectives are mainly used to describe or modify nouns and pronouns, indicating the nature, state, characteristics, or attributes of people or things; in user comments, they are reflected as the evaluation of relevant elements, which is consistent with the user concerns concern.

The second step is word frequency analysis. The purpose of word frequency analysis is to understand the characteristic word frequency and quickly grasp the comment hot spots in this situation (Chen et al., 2020). The nouns and adjectives after word segmentation are arranged in descending order. Based on the changing relationship between the word number and the proportion of the cumulative word frequency (Liu et al., 2016), the line is cut where the curve trend changes significantly, and the words whose cumulative word frequency proportion is hardly affected by the word number is eliminated.

The third step is word thematic analysis. Due to the differences in personal expression habits and context, thematic analysis is carried out on selected words to make the data more convergent (Oyebode et al., 2020). After the content analysis is carried out for the selected words, the nouns and adjectives are thematically analyzed, respectively, according to the semantic relevance (Ahn, 2021). Different product stimuli and user concerns are obtained, the product stimuli are further divided into object, activity, and identity, and the user concerns are further divided into goals, attitudes, and standards.

Finally, the “user concern–product stimulus” correlation is constructed. In order to conduct more scientific correlation calculations and comparisons, the normalized pointwise mutual information (NPMI) is introduced to characterize the specific correlation. In the informatics theory, mutual information (MI) is introduced to measure the relevance degree between two random variables (Yu et al., 2019). It is widely used in text analysis and natural language processing (NLP) (Qian and Cheung, 2019), especially for correlation calculation between texts, synonymy analysis, text classification, and feature matching (Zhang et al., 2019). The greater the mutual information value between variables, the stronger the correlation (Archer et al., 2013). The MI of two random variables X and Y is defined as follows:


(1) M⁢I⁢(X;Y)=∑x∈X,y∈Y⁢p⁢(x,y)⁢log⁢p⁢(x,y)/p⁢(x)⁢p⁢(y)

In Formula (1), *p* (x, y) refers to the probability of simultaneous occurrence of variables X and Y. In this study, it refers to the probability that a user concern and a product stimulus appear in a user comment at the same time, that is, the ratio of the simultaneous occurrence number between the two to the total comment number. *p* (x) refers to the probability of the occurrence of the variable x, that is, the ratio of the occurrence number of the variable x to the total comment number. *p* (y) can be obtained in the same way.

In order to further emphasize that a user concern and product stimulus appear in a user comment at the same time, pointwise mutual information (PMI) is introduced. Compared with the MI value, the PMI emphasizes the correlation of variables in a single event (Role and Nadif, 2011). The calculation formula of PMI is as follows:


(2) P⁢M⁢I⁢(X;Y)=log⁢p⁢(x,y)/p⁢(x)⁢p⁢(y)

Finally, in order to directly compare the PMI value, the normalized processing of pointwise mutual information is carried out to obtain the NPMI value (Bouma, 2009), which is used as the final value to characterize the correlation between a user concern and a product stimulus. The specific calculation process is as follows:


(3) N⁢P⁢M⁢I⁢(X;Y)=PMI/log⁢p⁢(x,y)

### Importing Comment Scores for Positive Experience Improvement Direction

When exploring positive user experience possibilities, the specific product use context and user group should be deeply understood in the specific product area. The user comment score reflects the overall satisfaction of users. The higher the score, the more obvious the positive emotion performance. At the same time, corresponding to the “appraisal” in the product emotion theory, it is the quantitative expression result of this parameter. In order to understand which “user concern—product stimulus” has the greatest impact on the user appraisal results, the “user concern–product stimulus” with strong correlation in section “Construct the Correlation of ‘User Concern—Product Stimulus’ Through Data Analysis” is taken as the research object, and *d* is defined as follows:


(4) $$\text{d}=\bar{V}\,\left(\mathrm{product}\,\mathrm{stimulus}\right)-\bar{V}\,\left(\mathrm{user}\,\mathrm{concern}\right)$$

In Formula (4), $\bar{V}$ (user concern) is the average user comment score involving a user concern, $\bar{V}$ (product stimulus) is the average user comment score involving a product stimulus, and d is the difference between $\bar{V}$ (user concern) and $\bar{V}$ (product stimulus). When *d* < 0, the average score of the product stimulus is less than that of the user concern, indicating that the product stimulus satisfies the user concern better. When d > 0, the average score of the product stimulus is greater than the user concern, revealing that the product stimulus does not satisfy the user concern. Therefore, a larger *d*-value represents greater improvement feasibility.

The NPMI value of “user concern–product stimulus” indicates the correlation strength. When the correlation is strong and the user concern is satisfied, it is easier to experience positive emotions and promote positive user experience, which represents the possibility.

The exploration of the positive user experience possibility would help promote positive improvement and iteration of products or services. However, in the design practice, feasibility should also be within the scope of practical discussion. The combination of them can be more conducive to the clarification of the design improvement direction.

## A Beverage Unmanned Retail Terminal Case

Based on the aforementioned discussion, this study takes the “Angel Orange” unmanned retail terminal as an example to discuss the positive user experience design. “Angel Orange” is one of the most widely distributed intelligent unmanned retail terminal brands in China, which sells freshly squeezed orange juice. It is distributed in major cities in China, and its user comment data can be publicly obtained on a comment website in China.

### Acquiring “Angel Orange” User Comment Data

In this example, the original user comment data set is obtained by using the web crawler technology, including the comment text of the user experience process (embodied as the unstructured statement) and the corresponding comment score, and the “Angel Orange” original user comment database is accessed.

### Constructing the “User Concern–Product Stimulus” Correlation Through Data Analysis

Before data analysis, the original user comment data are cleaned from text content identification and scoring screening. On the one hand, the unrecognizable conformity such as expression and illegal conformity is removed. On the other hand, the comment scores of this example include 0.5, 1, 1.5, 2, 2.5, 3, 3.5, 4, 4.5, and 5. User comment data with a score less than 3 are removed because, relatively speaking, the score of less than 3 is low, which belongs to negative evaluation, and the score between 3 and 5 is relatively high, which belongs to positive evaluation (Zong and Xia, 2020). This research studies positive user experience and pays more attention to the content of positive evaluation. Finally, 2,213 “Angel Orange” user comments were obtained.

**FIGURE 3:**
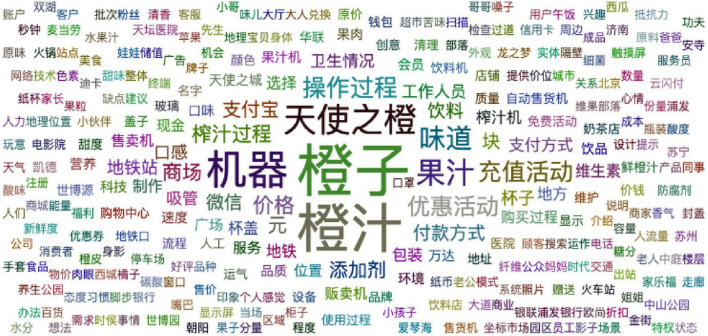
Noun cloud figure of “Angel Orange” unmanned retail terminal.

**FIGURE 4:**
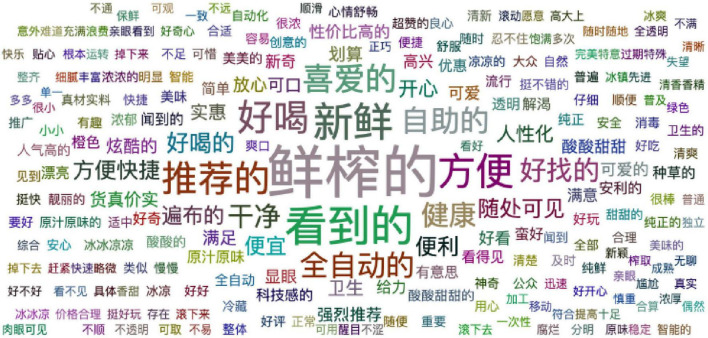
Adjective cloud figure of “Angel Orange” unmanned retail terminal.

Then, 2,213 user comments were imported into the online word segmentation platform Gooseeker, and 937 nouns and 561 adjectives were finally obtained. The word cloud figures of nouns and adjectives are shown in Figures 3, 4, respectively. A word cloud figure is a form of visual display of text information. The higher the word frequency, the larger the word displayed (Wang et al., 2020).

**FIGURE 5:**
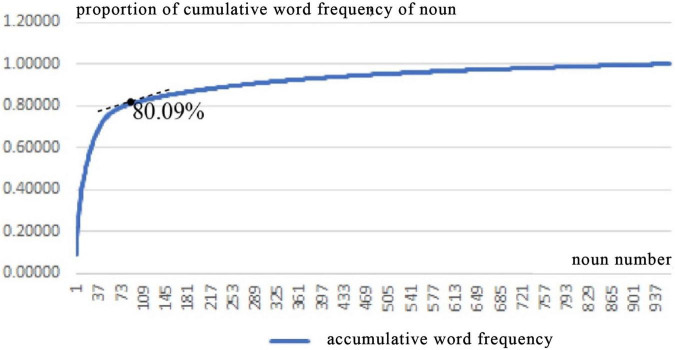
Relationship between noun number and proportion of cumulative word frequency.

**FIGURE 6:**
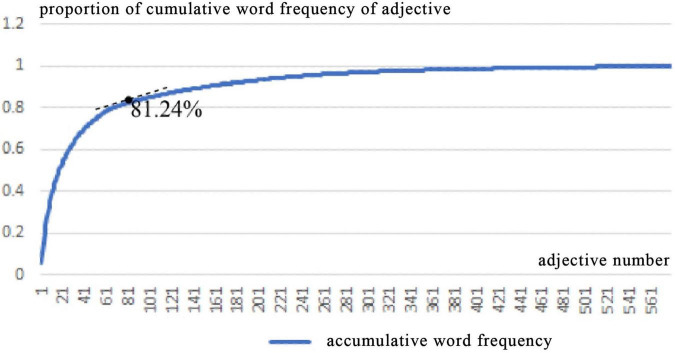
Relationship between adjective number and proportion of cumulative word frequency.

Second, in order to reduce manual intervention and ensure the authenticity and objectivity of the data as much as possible, words were sorted in descending order of frequency without synonym merging (Hu et al., 2013). Figure 5 shows the change curve of the noun number and the proportion of the accumulative word frequency. It can be seen that the proportion of the accumulative word frequency accounts for slightly less than 80%, and the curve trend has changed. A word frequency change of 10 was used as a slice, and finally, 82 nouns with a word frequency of 30 and above were selected, and the cumulative word frequency was 80.09%. Similarly, Figure 6 shows the change curve of the adjective number and the proportion of the accumulative word frequency. It can be seen that the cumulative word frequency is slightly higher than 80%, and the curve trend has changed. A word frequency change of 10 was used as a slice, and finally, 69 adjectives with a word frequency of 20 and above were selected, and the cumulative word frequency was 81.24%.

**FIGURE 7:**
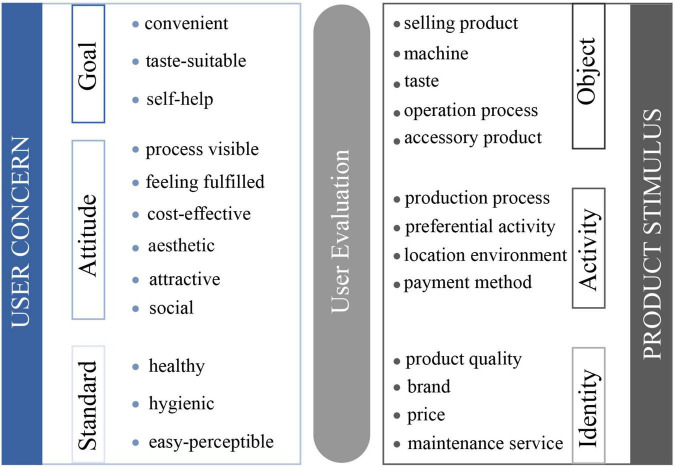
Division of user concerns and product stimuli.

Third, in order to further converge the data, 87 nouns and 75 adjectives were thematized. To ensure the reliability of content analysis results, each designer in this project was involved in the content analysis process and followed the following three principles in content analysis: (1) keep systematic and objective during content analysis; (2) content analysis is universal to the entire user comment document; and (3) principle applies to certain user comment documents (Vaismoradi et al., 2013). Table 1 describes a thematized example of nouns, that is, product stimuli. Table 2 describes a thematized example of adjectives, that is, user concerns. From the thematizing results of beverage unmanned retail terminals, 13 product stimuli, product quality, selling products, machine, brand, taste, operation process, preferential activities, price, production process, location environment, payment methods, ancillary products, and maintenance services, and 12 user concerns, healthy, process visible, convenient, taste-suitable, feeling fulfilled, self-service, hygienic, social, easy-perceptible, cost-effective, aesthetic, and attractive, were mentioned. The user concern categories and the product stimulus categories are listed in Tables 3, 4. Figure 7 describes the division of the 13 product stimuli and 12 categories of user concerns of “Angel Orange” based on the product emotion theory.

**TABLE 1:** Thematized example of nouns table.

Product stimulus	Noun tag words and frequency	Total
Product quality	Orange 1,826, additive 302, vitamin140, nutrition76…	2,527
Selling product	Orange juice1,688, juice710, beverage 70, drink 249…	2,747
Machine	Machine 1,174, juicer 143, vending machine 127, selling machine 105…	1,918
Brand	Angel Orange 887, angel city 61, brand 45, name 20…	1,013
Taste	Taste 592, flavor 237, texture94, sweetness94…	961
Operation process	Operation process 510, selection 156, purchasing process 120, use process 120…	919
Preferential activity	Promotions 440, recharge activity 294, member 75, free activity 49…	858
Price	Price 364, 15 yuan 307, original price35, selling price 32…	997
Production process	Production process 340, juicing process 147, production speed 75…	562
Location environment	Mall 339, subway station 227, place 136, palace 125…	1,300
Payment method	Alipay 294, WeChat 264, payment method 219, cash 103…	1,096
Accessory product	Cup 219, straw203, lid 167, package 150…	1,024
Maintenance service	Hygiene service 190, staff member 174, service 117, maintenance 45…	591

**TABLE 2:** Thematized example of adjectives table.

User concern	Adjective tag words and frequency	Total
Healthy	Freshly squeezed 816, fresh 649, healthy 364, safe 22…	1,581
Process visible	Witnessed 732, transparent 72, clear 27…	831
Convenient	Convenient 559, easy 66, convenient and fast 30, fast 243…	1,179
Taste-suitable	Tasty 551, delicious 163, sour and sweet 111, quench thirst 56…	1,215
Feeling fulfilled	Happy 184, humane 162, delightful 88, fulfilled 155…	725
Self-help	Self-help 440, automatic 379, intelligent 23…	842
Hygienic	Clean 343, hygienic 155, relieved 126…	624
Social	Recommended 357, shared 68, social 51…	481
Easy-perceptible	Easy-perceptible 339, easy-finding 227, pervasive 136, obvious 134 …	906
Cost-effective	Cheap 209, affordable 142, inexpensive 140, cost -effective 111…	794
Aesthetic	Cool 137, cute 117, good-looking 106, beautiful 62…	545
Attractive	Favorite 405, curious 63, popular 50, interesting 75, fashionable…	724

**TABLE 3:** Meaning of product stimulus.

Product stimulus	Meaning of product stimulus
Product quality	The quality and freshness of the drink
Selling product	Products sold by vending machines
Machine	Including the machine conditions of hardware and software
Brand	User’s awareness of products and product series
Taste	The hot, sour, sweetness, and sour attributes of the drink
Operation process	The operation required by the user to obtain the drink
Preferential activity	Various forms of promotional activities such as recharge and transaction gifts
Price	Price
Production process	Juicing process from oranges to orange juice
Location environment	The location and environment of the beverage machine
Payment method	The way to pay by yourself
accessory product	Accessories that assist users in a better experience
Maintenance service	Maintenance and adjustment of machines and interfaces, cleaning of internal juice extraction structure

**TABLE 4:** Results for extracted expectation.

User concern	The meaning of user concern
Healthy	The product is good for the body
Process visible	The production process can be seen
Convenient	How smoothly users can obtain products by themselves
Taste-suitable	The taste adapts to the individual and the environment
Feeling fulfilled	The user is happy with the whole experience process
Self-help	Users can obtain products completely independently
Hygienic	The machine and the juicing structure are clean
Social	Connection with others through self-service kiosks
Easy-perceptible	The machine is easily discovered and perceived by users
Cost-effective	The user’s expenditure is in line with the product acquisition
Aesthetic	Visual information such as machine appearance conforms to human perception
Attractive	The experience process meets user expectations and feels interesting

**FIGURE 8:**
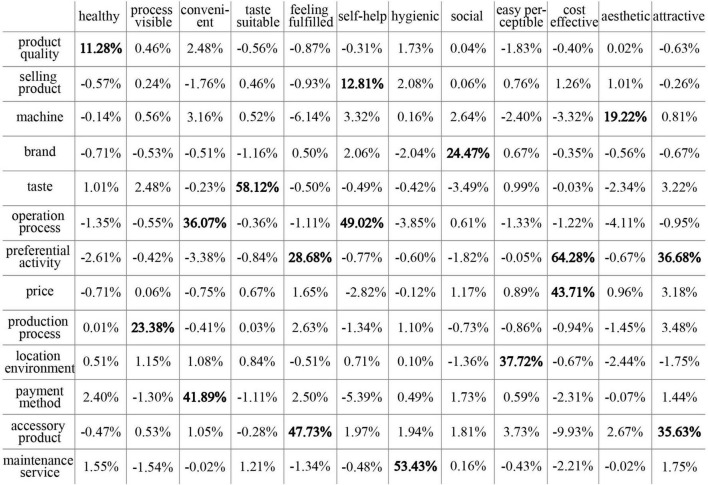
NPMI value between 12 user concerns and 13 product stimuli.

Based on Figure 2, using Formulas (1), (2), and (3), the correlation between the product stimulus and user concern is calculated through a Python program. Figure 8 shows the NPMI value of 13 product stimuli and 12 user concerns. “User concern–product stimulus” with an NPMI value greater than 10% was selected for the next discussion because a strong correlation could elicit positive emotions in the product emotion theory. The final 17 “user concern–product stimulus” pairs obtained were further studied. As shown in Figure 8, one user concern may have stronger relevance with more than one product stimulus.

**FIGURE 9:**
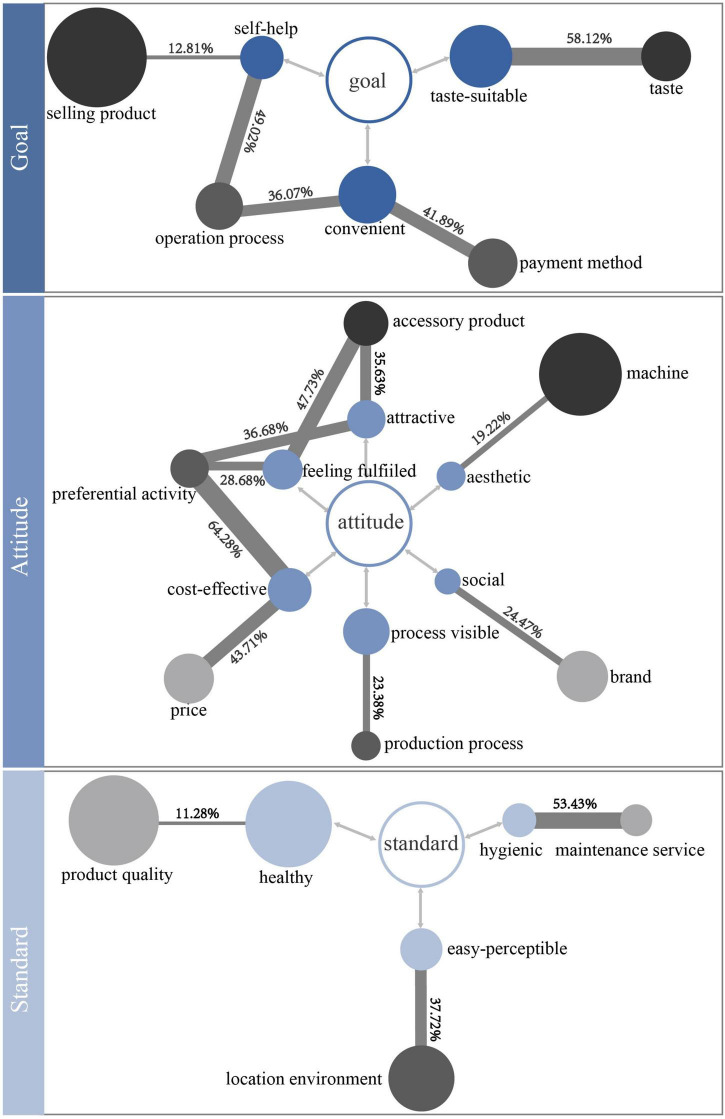
Three types of user concern correlation networks.

User concerns reveal what users want or aspire to, and product stimuli are the way to satisfy user concerns. Therefore, a better understanding of the relationship between user concerns and product stimuli is more conducive to exploring positive user experience possibilities. Then, the obtained data were converted into more intuitive graphics, which is divided into three parts according to the user concern types. As shown in Figure 9, gray represents three types of product stimuli; the circles with three different gray levels represent object, activity, and identity (from dark to light, respectively); and blue represents three types of user concerns, goal, attitude, and standard (from dark to light, respectively). The size of the circle corresponds to the frequency of the thematizing word; the larger the circle radius, the higher the frequency. The line thickness between the gray circle and blue circle indicates the “user concern–product stimulus” relevance strength; the thicker the line, the stronger the relevance. The value on the line indicates the specific NPMI value between the user concern and product stimulus. It can be clearly seen that the attitude of user concern in the relevance network is the most complicated. Attractive, feeling fulfilled, and cost-effective are frequently mentioned; the product stimuli related to attitude are the most extensive. The goal of user concerns involves three aspects: taste-suitable, self-service, and convenient. As for the standard of user concerns, healthy, hygiene, and easy-perceptible are mentioned. The commonalities and differences of the three relevance networks will be discussed in detail.

As mentioned earlier, attitude expresses the user’s concern to “pleasure” in the entire experience process. First, in the attitude relevance network, it is notable that preferential activity is highly correlated with the three categories of user concerns: attractive, feeling fulfilled, and cost-effective. It shows that preferential activity affects the user’s “pleasure” to a great extent. Preferential activity has the strongest relevance with cost-effective, but frequency is not the highest, indicating that it seems the most ideal to provide cost-effective preferential activity. Second, attractive, feeling fulfilled, and cost-effective are all related to the two categories of the product stimulus. Attractive is related to accessory product and preferential activity; feeling fulfilled is related to accessory product and preferential activity; cost-effective is related to price and preferential activity. This shows that one category of the user concern may require more than one category of the product stimulus to satisfy, and one category of the product stimulus may satisfy multiple categories of the user concern at the same time. Accessory product, preferential activity, and price are all the attributes or added values of products, but they affect whether users feel “pleasant” at a deeper level. Moreover, machine is the most mentioned product stimulus, but its relevance to aesthetic is the lowest in the attitude relevance network. This means that the user’s concern to machine is not significant in the aesthetic aspect. Brand is related to the social aspect, indicating that users hope to establish a connection between themselves and others through the influence of the brand. The relevance between the production process and the process visible is also reflected in this network. This shows that the user wants to see the production process of the product while waiting. It can be concluded from this relevance network that “pleasure” in the experience process is very important. Attitude involves a relatively wide range; it may be better if more aspects of the product stimulus match the user’s personal preferences.

Goal and standard are also very important. In the “goal” relevance network, selling product is the most mentioned product stimulus, but its relevance to self-service is the lowest. This is a matter of course that users would unconsciously express the objects evaluated when making statements. Both convenient and self-service are related to two categories of the product stimulus, showing that the convenience and self-help of the product acquisition process are not determined by only one category of the product stimulus. In the “standard” relevance network, healthy, hygienic, and easy-perceptible are the user concerns mentioned. Product quality, which is the most mentioned product stimulus by users, is related to healthy. The product stimuli related to the standard are the least, which indicates that it is not the type that users are most concerned about.

In summary, a general comprehension of different concerns has been obtained, among which attitude is the most mentioned by users. However, the positive experience possibilities that can give guidance on design practice have not yet been proposed.

### Import Comment Scores for Positive User Experience Interval Division

**FIGURE 10:**
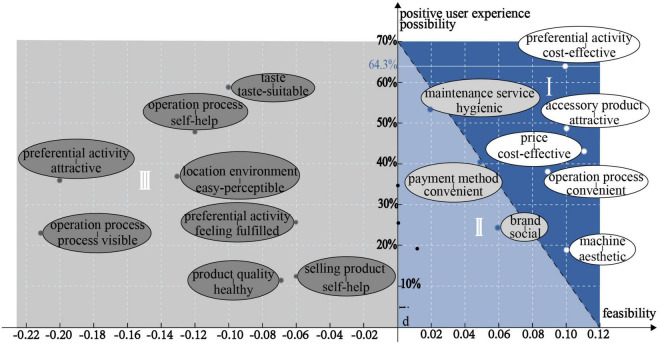
Division of positive user experience interval.

It is obvious that more than one product stimulus and user concern may be mentioned in a user comment. In order to understand which “user concern–product stimulus” pairs have the greatest impact on the user appraisal results, the 17 “user concern–product stimulus” pairs obtained in the previous study are regarded as the research objects. According to Formula (4) and NPMI, the positive user experience interval division is shown in Figure 10.

Unmanned retail terminals provide services to the public, so it is necessary to understand various positive emotions to explore the multiple positive experience possibilities. User concerns could be regarded as the user needs and expectations to a certain extent but cannot be improved by designers, whereas product stimuli could be improved and enhanced. As shown in Figure 10, when the “user concern–product stimulus” pairs are located in zone 3, d < 0, the user concerns are already satisfied and the positive emotions are obvious, so the feasibility of subsequent improvements is relatively low. When the pairs are located in zone 1 or 2, d > 0, the user concerns are not satisfied, so the product stimuli are worthy of improvement and enhancement. Furthermore, when the pairs are located in zone 1, d and NPMI are higher, and the feasibility and possibility values are relatively high, so the product stimuli should be prioritized for improvement.

## Discussion

With the continuous development of disciplines such as user experience and the upgrading and transformation of personal needs and expectations, designers need new possibilities to create a positive user experience rather than just solving existing problems. This is both a challenge and an opportunity. Based on the product emotion theory, the contribution of this study can be summarized as follows:

(1) Starting from the user “positive experience” in specific situations, it emphasizes arousing positive emotions in the experience process so as to improve the positive user experience.
(2) Based on the product emotion theory, this study understands the user experience from the user and the product and defines the product stimuli that affect the user experience and the corresponding user concern. In particular, when used to guide design practice, “user concern—product stimulus” is taken as the research object rather than just focusing on what the product provides to users.
(3) The product emotion theory is applied to the analysis process of positive “user comment,” which is embodied in establishing a mapping relationship between user concerns, product stimuli in the product emotion theory, and the comment text. The factors affecting the positive user experience are analyzed from multiple levels. According to the comment score, the positive experience interval is divided, the improvement direction is put forward, and the feasibility is verified by an example.

The authors point out that this study can bring inspiration to a positive user experience design or guide the direction to a certain extent. At the same time, more in-depth research should be carried out in future. For example, the positive user experience feasibility should be verified from multiple practice fields, and more scientific and rigorous methods should be sought out to prioritize the improvement of each product stimulus.

## Conclusion

The results show that user concerns of unmanned retail terminals are reflected not only in practical aspects but also in hedonic aspects. From the correlation network, attitude, which is mentioned the most, indicates that the pleasure of the experience process is highly important to a positive user experience. Machine, accessory product, brand, preferential activity, production process, and price are all related to the pleasure of the user experience process. Machine and accessory product are the objects of the product stimulus, and users could directly interact with them to satisfy pleasure. Preferential activity and production process are the activities that can promote the interaction between users and products to satisfy pleasure. Price and brand are the identities of the product stimulus, which can promote users’ self-perception to satisfy pleasure.

**FIGURE 11:**
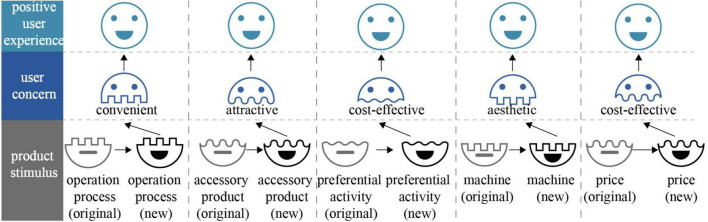
Improvement direction around “Angel Orange” unmanned retail terminal.

Regarding the design improvement, the “user concern–product stimulus” pairs located in zone 1 are put forward, and five specific improvement directions are depicted in Figure 11. User concerns cannot be improved, and product stimuli need to be improved. The product stimulus should be improved to the corresponding user concern-implied direction. Specifically, the operation process should be as convenient as possible. Providing more attractive accessory products can elicit a positive user experience. Preferential activity and price should be more cost-effective. The machine should meet the user’s aesthetics. These results are in line with the retail situation of “Angel Orange.” This means that design activities can be interposed for improving product stimuli to meet user concerns to elicit a positive user experience.

Designers should make it clear that focusing merely on the problem may only solve the existing problems and cannot bring new possibilities. After making it clear that arousing positive emotions is conducive to improving a positive user experience, this study starts from a “positive experience” story. The mapping relationship between positive user comments and the parameters of the product emotion theory is established. As evident from empirical results, findings based on positive user comments are fruitful. More attractive, more convenient, more cost-effective, and more aesthetic “Angel Orange” unmanned retail terminal’s positive user experience design possibilities are proposed.

## Data Availability Statement

The original contributions presented in this study are included in the article/supplementary material, further inquiries can be directed to the corresponding author/s.

## Author Contributions

EC: ideas, formulation and evolution of overarching research goals and aims, methodology, and revision. YD: writing—original draft preparation, creation (including substantive translation), and logical combining of the full text. JJ: maintenance of research data (including software code for initial use and later reuse). HP: review and revision. WH: important contributions to this revision. All authors contributed to the article and approved the submitted version.

## Conflict of Interest

The authors declare that the research was conducted in the absence of any commercial or financial relationships that could be construed as a potential conflict of interest.

## Publisher’s Note

All claims expressed in this article are solely those of the authors and do not necessarily represent those of their affiliated organizations, or those of the publisher, the editors and the reviewers. Any product that may be evaluated in this article, or claim that may be made by its manufacturer, is not guaranteed or endorsed by the publisher.
